# The prevalence and genotype distribution of human papillomavirus in central Fujian Province during the COVID-19 pandemic

**DOI:** 10.1186/s12985-024-02393-z

**Published:** 2024-06-05

**Authors:** Boxi Lin, Fu Zhang, Fang Liu, Lihua Huang, Shanzhen Xie, Qing Lin, Jilai Liu

**Affiliations:** 1 Department of Clinical Laboratory, People’s Hospital Affiliated of Fujian University of Traditional Chinese Medicine, Fuzhou, 350004 China; 2 Department of Preventive Medicine, People’s Hospital Affiliated of Fujian University of Traditional Chinese Medicine, Fuzhou, 350004 China

**Keywords:** Human papillomavirus, COVID-19 pandemic, Cervical cancer, Prevalence, Genotype

## Abstract

**Background:**

Global human activities were significantly impacted by the emergence of the coronavirus disease 2019 (COVID-19) pandemic caused by the 2019 novel coronavirus. This study aimed to investigate the prevalence and genotype distribution of HPV infection in Central Fujian Province during the pandemic.

**Methods:**

Cervical samples were collected from 21,612 outpatients and 12,664 females who underwent physical examinations and HPV screening at the People’s Hospital of Fujian Province in Fuzhou from April 2020 to April 2023. HPV detection and genotyping were conducted using PCR hybridization.

**Results:**

The overall HPV infection rate was 16.1% during the COVID-19 pandemic, with the outpatient group exhibiting a greater infection rate (19.0%) than did the healthy group (12.3%). The top five high-risk HPV (HR-HPV) genotypes in both groups were HPV52, HPV53, HPV58, HPV16, and HPV51. Additionally, HPV81 and HPV43 were the two most common low-risk HPV (LR-HPV) genotypes in the patient group, while HPV81 and HPV42 were the two most common LR-HPV genotypes in the healthy group. The highest prevalence of HPV infection was observed in individuals aged ≤ 24 years (28.4%, 95% CI 25.9–30.9), followed by those aged ≥ 55 years (23.6%, 95% CI 21.6–24.7) and other age groups. The prevalence decreased from 23.0% (95% CI 22.4–23.7) in 2018–2019 to 13.8% (95% CI 12.0-15.5) in 2023.

**Conclusion:**

This study provides valuable insights into the prevalence and genotypes of HPV infection in the female population of Central Fujian Province from 2020 to 2023. The findings indicate that the prevalence of HPV infection in Central Fujian Province remains relatively low compared to the national average. Furthermore, the prevalence of HPV decreased during the COVID-19 pandemic; however, as the pandemic waned, there was potential for an increase in HPV infection rates. Therefore, it is crucial to strengthen HPV screening and vaccination strategies to prevent the potential spread of HPV.

## Introduction

According to GLOBOCAN 2020 statistics, cervical cancer ranks as the fourth most commonly diagnosed cancer and the fourth leading cause of cancer-related deaths in women, with an estimated 604,000 new cases and 342,000 deaths globally in 2020 [1]. In response, the World Health Organization (WHO) called for a global initiative in May 2018 to eliminate cervical cancer as a public health concern [2]. Risk factors for cervical cancer include sexually transmitted infections (such as HIV and *Chlamydia trachomatis*), smoking, a greater number of childbirths, and long-term use of oral contraceptives [3]. Additionally, cervical cancer burden has been associated with the Human Development Index (HDI) [4], and disparities exist even within the same country, as evidenced by the fact that cervical cancer mortality among women in poor counties in the United States is twice that among women in affluent counties [5].

Human papillomavirus (HPV) is a major etiological factor in almost all cases of cervical cancer and is also responsible for a significant portion of other anogenital and oropharyngeal cancers [6]. There are more than 200 established genotypes of HPV, which belong to 49 species in five genera [7]. These genotypes can be categorized into high-risk (HR) and low-risk (LR) types based on their carcinogenic potential. Epidemiological studies and mechanistic evidence have revealed that HPV16, 18, 31, 33, 35, 39, 45, 51, 52, 56, 58, and 59 are carcinogenic, while HPV68 is considered potentially carcinogenic [6]. Currently, HPV vaccination and screening are the primary strategies for preventing cervical cancer. The WHO has established an ambitious target to eradicate cervical cancer by achieving 90% vaccination coverage for girls by age 15 and screening 70% of women at ages 35 and 45 [8].

The emergence of the coronavirus disease 2019 (COVID-19) pandemic led to profound changes in human activity. Strict lockdowns, travel restrictions, and limited social interactions have had a negative impact on cancer screening. Hence, it is crucial to evaluate the potential impact of the WHO’s ambitious initiative. This study conducted a comprehensive survey of the prevalence and genotype distribution of HPV in Central Fujian Province during the COVID-19 pandemic. The results can shed light on the impact of the COVID-19 pandemic on HPV infection rates and provide a basis for improving cervical cancer prevention and HPV screening strategies in Central Fujian after the pandemic.

## Materials and methods

### Study population

The study population for this research consisted of a total of 37,836 female patients who underwent HPV screening at the People’s Hospital of Fujian Province from April 2020 to April 2023. The median age of the participants was 42 years, with a range from 14 to 94 years. Among these subjects, 21,612 visited the clinic for medical care related to various gynecological issues, while 16,224 were seeking physical examinations. For comparison, the analysis included data from 27,993 female individuals, collected between 2018 and 2019, comprising 14,168 patients and 13,825 physical examinations. Duplicate data from the same patient were removed, leaving only the first screening result. The study was approved by the Institutional Medical Ethics Review Board of People’s Hospital Affiliated with Fujian University of Traditional Chinese Medicine, Fuzhou, China (Grant No. 2023-60-01).

### Sample collection and HPV genotyping

Cervical samples were collected from patients using a cytobrush, preserved in a special buffer solution and stored at 2–8 °C for less than 48 h. The cervical samples were vortexed and mixed before DNA extraction. Nucleic acid extraction reagent and HPV22 genotyping assay reagent (Shanghai Tellgen Co., Ltd.) were used for DNA extraction and HPV genotyping, respectively. Amplification and hybridization were performed using an ABI-7500 fluorescence quantitative PCR instrument and a Luminex-200 hybridization system. The assay system was capable of detecting 16 high-risk HPV types (16.18.31.33.35.39.45.51.52.53.56.58.59.66.68.82) and 6 low-risk HPV types (6.11.42.43.81.83). The procedures were strictly followed according to the instructions.

### Statistical analysis

For statistical analysis, all results were imported into EXCEL and sorted by sample source (outpatient and physical examination), age group (≤ 24, 25–34, 35–44, 45–54, ≥ 55 years), and year (2020, 2021, 2022, 2023). Binomial distribution analysis was utilized to calculate the 95% confidence intervals (95% CIs). The chi-square test (χ2 test) was used to assess whether there was a statistically significant difference in HPV infection rates among different groups, and the pairwise chi-square test (pairwise χ2 test) was used to compare whether there were significant differences in the infection rates of different genotypes across groups. Two-sided *P* values less than 0.05 were considered to indicate statistical significance. In cases where one or more of the expected numbers were ≤ 5 or the *P* value was close to 0.05, Fisher’s exact test was applied. All the statistical analyses were conducted using SPSS 26.0.

## Results

### The overall prevalence of HPV infection during COVID-19

In total, 37,836 subjects were included in the study, with 6,083 (16.1%, 95% CI 15.7–16.4) testing positive for HPV DNA. When stratified by group, the prevalence of HPV DNA was 19.0% (95% CI 18.4–19.4) in the outpatient group and 12.3% (95% CI 11.8–12.8) in the healthy group (Table 1). The difference in prevalence between the two groups was found to be statistically significant (χ2 = 299.9, *P* < 0.005).

**Table 1:** Prevalence rate of HPV genotypes in the study population (2020–2023)

HPV genotype	Patients		Healthy women		χ2	*P* value
	No.	Prevalence % (95% CI)	No.	Prevalence % (95% CI)		
**Any type**	4087	19.0 (18.4-19.4)	1996	12.3(11.8-12.8)	299.9	<0.005
**Single infections**	3145	15.0 (14.1-15.0)	1574	9.7(9.2-10.2)	199.7	<0.005
**Multiple infections**	942	4.4 (4.1-4.6)	422	2.6(2.4-2.8)	82.4	<0.005
**HPV52**	954	4.4 (4.1-4.7)	453	2.8 (2.5-3.0)	68	<0.005
**HPV53**	505	2.3 (2.2-2.6)	250	1.5 (1.3-1.7)	30	<0.025
**HPV58**	441	2.0 (1.8-2.2)	176	1.1 (0.9-1.2)	52.8	<0.01
**HPV16**	417	1.9 (1.7-2.1)	158	1.0 (0.8-1.1)	56.6	<0.01
**HPV51**	273	1.3 (1.1-1.4)	153	0.9 (0.8-1.1)	8.5	<0.05
**HPV39**	242	1.1 (1.0-1.3)	92	0.6 (0.5-0.7)	32.3	<0.01
**HPV59**	237	1.1 (1.0-1.2)	117	0.7 (0.6-0.9)	14.1	<0.025
**HPV56**	236	1.1 (1.0-1.2)	113	0.7 (0.6-0.8)	15.9	<0.025
**HPV18**	233	1.1 (0.9-1.2)	101	0.6 (0.5-0.7)	22	<0.025
**HPV68**	190	0.9 (0.8-1.1)	100	0.6 (0.5-0.7)	8.4	<0.05
**HPV66**	177	0.8 (0.7-0.9)	84	0.5 (0.4-0.6)	12.2	<0.025
**HPV33**	112	0.5 (0.4-0.6)	61	0.4 (0.3-0.5)	4.1	>0.05
**HPV35**	95	0.5 (0.4-0.5)	24	0.1 (0.1-0.2)	25.1	<0.025
**HPV31**	88	0.4 (0.3-0.5)	44	0.3 (0.2-0.4)	4.9	>0.05
**HPV45**	63	0.3 (0.2-0.4)	24	0.1 (0.1-0.2)	8.3	<0.05
**HPV82**	47	0.2 (0.2-0.3)	16	0 (0.1-0.1)	7.9	<0.05
**HPV81**	336	1.6 (1.4-1.7)	230	1.4 (1.2-1.6)	1.2	>0.1
**HPV43**	208	1.0 (0.8-1.1)	117	0.7 (0.6-0.9)	6.3	>0.05
**HPV6**	188	0.9 (0.7-1.0)	56	0.3 (0.3-0.4)	39.8	<0.01
**HPV42**	176	0.8 (0.7-0.9)	127	0.8 (0.6-0.9)	0.1	>0.1
**HPV11**	99	0.5 (0.4-0.5)	30	0.2 (0.1-0.3)	20.4	<0.025
**HPV83**	47	0.2 (0.2-0.3)	19	0.1 (0.1-0.2)	5.4	>0.05

Underlining indicates no significant difference between the two groups

Among the HPV DNA-positive patients, 77.0% (95% CI 75.7–78.2) were infected with a single HPV type, 17.3% (95% CI 16.1–18.4) were infected with two HPV types, 4.2% (95% CI 3.5–4.8) were infected with three HPV types, and 1.1% (95% CI 0.7–1.4) were infected with four HPV types. In the healthy group, 78.9% (95% CI 77.1–80.6) were infected with a single HPV type, 16.5% (95% CI 14.9–18.1) were infected with two HPV types, 3.5% (95% CI 2.7–4.3) were infected with three HPV types, and 0.9% (95% CI 0.4–1.3) were infected with four HPV types. However, the rates of single and multiple HPV infections were not significantly different between the two groups (Table 2).

**Table 2:** Prevalence of single and multiple HPV genotype infections in the study population (2020–2023)

Infection types	Patients		Healthy women		χ2	*P* value
	Positive no.	% for all infected patients (95% CI)	Positive no.	% for all infected patients (95% CI)		
**Single infections**	3145	77.0(75.7-78.2)	1574	78.9(77.1-80.6)	2.8	>0.05
**Double infections**	706	17.3(16.1-18.4)	329	16.5(14.9-18.1)	0.6	>0.1
**Triple infections**	170	4.2(3.5-4.8)	69	3.5(2.7-4.3)	1.8	>0.1
**Quadruple infections**	43	1.1(0.7-1.4)	17	0.9(0.4-1.3)	0.6	>0.1
**Quintuple infections**	16	0.4(0.2-0.6)	5	0.3(0.0-0.5)	0/8	>0.1
**Sextuple infections**	5	0.1(0.0-0.2)	1	0.0(0.0-0.1)	0/7	>0.1
**Septuple infections**	1	0.0(0.0-0.1)	1	0.0(0.0-0.1)	0.3	>0.1
**Eightfold infections**	1	0.0(0.0-0.1)	NA	NA	NA	NA

### Genotype-specific prevalence of HPV infection during COVID-19

In this study, 22 different genotypes of HPV were identified, comprising 16 high-risk HPV genotypes and 6 low-risk HPV genotypes. The percentages of HR-HPV-positive patients were 16.3% (95% CI 15.8–16.8) and 10.5% (95% CI 10.1–11.0) for the two groups, respectively.

**Fig. 1:**
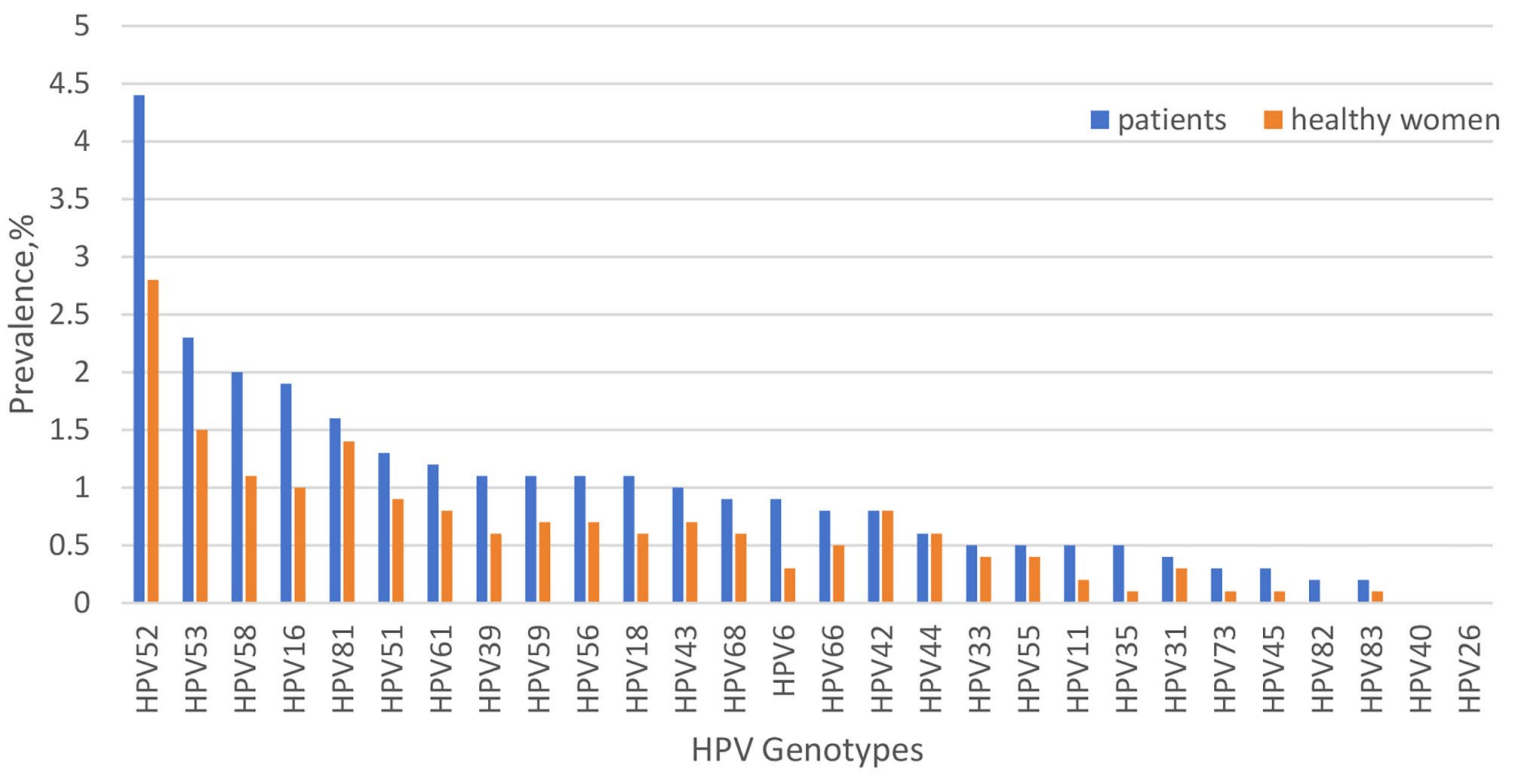
Prevalence of HPV genotypes in 21,612 patients and 16,224 healthy individuals

The top five HR-HPV genotypes in both groups were HPV52, HPV53, HPV58, HPV16, and HPV51 (Table 1; Fig. 1). Additionally, the two most common LR-HPV genotypes in the patient group were HPV81 and HPV43, while in the healthy group, they were HPV81 and HPV42. Significant differences in infection rates between the two groups were observed for most genotypes, with the exception of HPV81, HPV43, HPV42, HPV33, HPV31, and HPV83.

### Age-specific prevalence of HPV infection during COVID-19

In this study, all subjects were categorized into five age groups: ≤24, 25–34, 35–44, 45–54, and ≥ 55 years. The majority of subjects in both the patient and healthy groups fell within the 35–44 and 45–54 age categories.

In the patient group, the highest prevalence of HPV infection was observed in the ≤ 24 years age group, with a rate of 28.4% (95% CI 25.9–30.9), followed by the ≥ 55 years age group, with a rate of 23.6% (95% CI 21.6–24.7). No significant differences were found in infection rates among the 25–34, 35–44, and 45–54 age groups (Table 3).

**Table 3:** The infection rates and genotype distributions of HPV in age-specific groups

HPV genotypes	Years (Patients)					Years (Healthy women)				
	≤24 *N* (%)	25-34 *N* (%)	35-44 *N* (%)	45-54 *N* (%)	≥55 *N* (%)	≤24 *N* (%)	25-34 *N* (%)	35-44 *N* (%)	45-54 *N* (%)	≥55 *N* (%)
**Sample size**	1252(5.8)	5115(23.7)	6266(29.0)	6190(28.6)	2789(12.9)	186(1.1)	3619(22.3)	5504(33.9)	4999(30.8)	1916(11.8)
**Positive**	355(28.4)^a*^	965(18.9)^b^	1059(16.9)^b^	1062(17.2)^b^	646(23.2)^c^	34(18.3)^a^	395(10.9)^b^.	605(11.0)^b^	641(12.8)^c^	321(16.8)^a^
**Single infections**	244(19.5)^a^	727(14.2)^b^	850(13.6)^b^	854(13.8)^b^	470(17.2)^c^	26(14.0)^a.b^	312(8.6)^b^	493(9.0)^b^	503(10.1)^b^	240(12.5)^a^
**Multiple infections**	111(8.9)^a^	238(4.7)^b^	209(3.3)^c^	208(3.4)^c^	176(6.3)^d^	8(4.3)^a.b^	83(2.3)^b^	112(2.0)^b^	138(2.8)^b^	81(4.2)^a^
**HPV16**	57(4.6)^a^	97(1.9)^b.c^	89(1.4)^c^	107(1.7)^b.c^	67(2.4)^b^	NA	38(1.1)^a^	41(0.7)^a^	52(1.0)^a^	27(1.4)^a^
**HPV18**	22(1.8)^a^	56(1.1)^ab^	62(1.0)^b^	52(0.8)^a^	41(1.5)^b^	5(2.7)^a^	18(0.5)^b^	31(0.6)^b^	35(0.7)^b^	12(0.6)^b^
**HPV31**	3(0.2)^a^	21(0.4)^a^	30(0.5)^a^	22(0.4)^a^	12(0.4)^a^	1(0.5)^a.b^	16(0.4)^b^	6(0.1)^a^	14(0.3)^a.b^	7(0.4)^a.b^
**HPV33**	15(1.2)^a^	26(0.5)^a.b^	24(0.4)^b^	25(0.4)^b^	22(0.8)^a.b^	1(0.5)^a.b^	13(0.4)^a.b^	19(0.3)^b^	11(0.2)^b^	17(0.9)^a^
**HPV35**	11(0.9)^a^	18(0.4)^a.b^	32(0.5)^a.b^	16(0.3)^b^	18(0.6)^a.b^	NA	1(0.0)^a^	11(0.2)^a^	7(0.1)^a^	5(0.3)^a^
**HPV39**	19(1.5)^a^	63(1.2)^a^	59(0.9)^a^	72(1.2)^a^	29(1.0)^a^	3(1.6)^a^	20(0.6)^a^	26(0.5)^a^	31(0.6)^a^	12(0.6)^a^
**HPV45**	5(0.4)^a^	8(0.2)^a^	24(0.4)^a^	13(0.2)^a^	13(0.5)^a^	1(0.5)^a^	6(0.2)^a^	4(0.1)^a^	11(0.2)^a^	2(0.1)^a^
**HPV51**	37(3.0)^a^	80(1.6)^b^	65(1.0)^b^	65(1.1)^b^	26(0.9)^b^	4(2.2)^a^	35(1.0)^a^	50(0.9)^a^	49(1.0)^a^	15(0.8)^a^
**HPV52**	67(5.4)^a.b^	240(4.7)^b^	249(4.0)^b.c^	212(3.4)^c^	186(6.7)^a^	11(5.9)^a^	85(2.3)^b^	135(2.5)^b^	143(2.9)^b^	79(4.1)^a^
**HPV53**	35(2.8)^a.b.c^	87(1.7)^c^	126(2.0)^b.c^	156(2.5)^b^	101(3.6)^a^	2(1.1)^a.b^	39(1.1)^b^	67(1.2)^b^	100(2.0)^a^	42(2.2)^a^
**HPV56**	19(1.5)^a^	57(1.1)^a^	67(1.1)^a^	61(1.0)^a^	32(1.1)^a^	5(2.7)^a^	18(0.5)^b^	32(0.6)^b^	40(0.8)^a.b^	18(0.9)^a.b^
**HPV58**	39(3.1)^a.b^	102(2.0)^b.c^	101(1.6)^c^	115(1.9)^c^	84(3.0)^a^	2(1.1)^a.b^	37(1.0)^b^	44(0.8)^b^	52(1.0)^b^	41(2.1)^a^
**HPV59**	29(2.3)^a^	52(1.0)^b^	66(1.1)^b^	61(1.0)^b^	29(1.0)^b^	4(2.2)^a^	35(1.0)^a^	30(0.5)^a^	32(0.6)^a^	16(0.8)^a^
**HPV66**	19(1.5)^a^	48(0.9)^a.b^	48(0.8)^a.b^	36(0.6)^b^	26(0.9)^a.b^	NA	15(0.4)^a^	37(0.7)^a^	17(0.3)^a^	15(0.8)^a^
**HPV68**	14(1.1)^a^	50(1.0)^a^	54(0.9)^a^	40(0.6)^a^	32(1.1)^a^	2(1.1)^a^	26(0.7)^a^	26(0.5)^a^	30(0.6)^a^	16(0.8)^a^
**HPV82**	14(1.1)^a^	18(0.4)^b^	9(0.1)^b.c^	2(0.0)^c^	4(0.1)^b.c^	NA	4(0.1)^a^	6(0.1)^a^	4(0.1)^a^	2(0.1)^a^
**HPV6**	50(4.0)^a^	49(1.0)^b^	29(0.5)^c^	35(0.6)^b.c^	25(0.9)^b.c^	1(0.5)^a^	6(0.2)^a^	18(0.3)^a^	24(0.5)^a^	7(0.4)^a^
**HPV11**	23(1.8)^a^	30(0.6)^b^	19(0.3)^b^	16(0.3)^b^	11(0.4)^b^	1(0.5)^a^	4(0.1)^a^	9(0.2)^a^	8(0.2)^a^	8(0.4)^a^
**HPV42**	16(1.3)^a.b^	39(0.8)^a.b^	40(0.6)^b^	46(0.7)^a.b^	35(1.3)^a^	1(0.5)^a^	21(0.6)^a^	40(0.7)^a^	44(0.9)^a^	21(1.1)^a^
**HPV43**	23(1.8)^a^	58(1.1)^a^	39(0.6)^b^	59(1.0)^a.b^	29(1.0)^a.b^	3(1.6)^a^	23(0.6)^a^	40(0.7)^a^	30(0.6)^a^	21(1.1)^a^
**HPV81**	13(1.0)^a^	78(1.5)^a^	75(1.2)^a^	102(1.6)^a.b^	68(2.4)^b^	4(2.2)^a.b^	46(1.3)^a.b^	64(1.2)^b^	76(1.5)^a.b^	40(2.1)^a^
**HPV83**	1(0.1)^a^	8(0.2)^a^	12(0.2)^a^	17(0.3)^a^	9(0.3)^a^	NA	3(0.1)^a^	5(0.1)^a^	7(0.1)^a^	4(0.2)^a^

*Each subscript letter (a, b, c, d) denotes a subset of age categories whose column proportions do not differ significantly from each other at the 0.05 level

### Year-specific incidence of HPV infection

In total, 27,993 subjects whose data were collected prior to the emergence of COVID-19 (2018–2019) were included in the study, among whom 5,436 (19.4%, 95% CI 19.0-19.9) tested positive for HPV DNA. This research investigated the prevalence of HPV infection in Central Fujian Province before and during the COVID-19 pandemic and revealed a decrease in the overall HPV infection rate. The prevalence of HPV infection did not significantly differ between the prepandemic period and 2020–2021, but a notable disparity was evident during 2022–2023. The prevalence decreased from 23.0% (95% CI 22.4–23.7) in 2018–2019 to 13.8% (95% CI 12.0-15.5) in 2023.

Further analysis revealed specific changes in the prevalence rates of different HPV genotypes over the study period. In the patient group, the prevalence rates of HPV42, 51, 52, 68 and 81 decreased from 2018 to 2023, while those of HPV11, 39, 43, 45, 59, 82, and 83 showed minimal changes.

In the healthy group, the prevalence rates of HPV42, 51, 52, 68, and 81 decreased from 2018 to 2023, while those of HPV11, 39, 59, and 83 remained stable over the same period (Table 4).

**Table 4:** The infection rates and genotype distributions of HPV in year-specific groups

HPV genotypes	Years (Patients)					Years (Healthy women)					
	2018–2019 *N* (%)	2020 *N* (%)	2021 *N* (%)	2022 *N* (%)	2023 *N* (%)	2018-2019 *N* (%)	2020 *N* (%)	2021 *N* (%)	2022 *N* (%)	2023 *N* (%)	
**Sample size**	14,168	4153	9656	6254	1549	13,825	4726	4148	5785	1565	
**Positive**	3265(23.0)a	951(22.9)a	2131(22.1)a	792(12.7)b	213(13.8)b	2171(15.7)a	727(15.4)a	699(16.9)a	436(7.5)b	134(8.6)b	
**Single infections**	2450(17.3)a	696(16.8)a	1615(16.7)a	658(10.5)b	176(11.4)b	1723(12.5)a	557(11.8)a	527(12.7)a	373(6.4)b	117(7.5)b	
**Multiple infections**	815(5.7)a	255(6.1)a	516(5.3)a	134(2.1)b	37(2.4)b	448(3.2)a	170(3.6)a	172(4.1)a	63(1.1)b	17(1.1)b	
**HPV16**	408(2.9)a	95(2.3)a.b	215(2.2)b	84(1.3)c	23(1.5)b.c	201(1.5)a	47(1.0)a.b	67(1.6)a	40(0.7)b.c	4(0.3)c	
**HPV18**	193(1.4)a	49(1.2)a	130(1.3)a	41(0.7)b	13(0.8)a.b	127(0.9)a	40(0.8)a	34(0.8)a	20(0.3)b	7(0.4)a.b	
**HPV31**	96(0.7)a	28(0.7)a	51(0.5)a	5(0.1)b	4(0.3)a.b	49(0.4)a	16(0.3)a	21(0.5)a	5(0.1)b	2(0.1)a.b	
**HPV33**	139(1.0)a	36(0.9)a.b	62(0.6)b	14(0.2)c	NA	75(0.5)a	28(0.6)a	26(0.6)a	5(0.1)b	2(0.1)a.b	
**HPV35**	65(0.5)a.b	19(0.5)a.b	56(0.6)b	17(0.3)a	3(0.2)a.b	34(0.2)a	7(0.1)a.b	15(0.4)a	2(0.0)b	NA	
**HPV39**	125(0.9)a	39(0.9)a	111(1.2)a	73(1.2)a	19(1.2)a	97(0.7)a	25(0.5)a	30(0.7)a	34(0.6)a	3(0.2)a	
**HPV45**	60(0.4)a	11(0.3)a	35(0.4)a	12(0.2)a	5(0.3)a	46(0.3)a	7(0.1)a.b	12(0.3)a.b	4(0.1)b	1(0.1)a.b	
**HPV51**	288(2.0)a	85(2.0)a.b	146(1.5)b	34(0.5)c	8(0.5)c	187(1.4)a	64(1.4)a	60(1.4)a	21(0.4)b	8(0.5)b	
**HPV52**	709(5.0)a	218(5.2)a	519(5.4)a	165(2.6)b	52(3.4)b	464(3.4)a	163(3.4)a	139(3.4)a	121(2.1)b	30(1.9)b	
**HPV53**	352(2.5)a	116(2.8)a	257(2.7)a	107(1.7)b	25(1.6)a.b	237(1.7)a	87(1.8)a	86(2.1)a	62(1.1)b	15(1.0)b	
**HPV56**	151(1.1)a.b	53(1.3)a.b	128(1.3)b	44(0.7)c	11(0.7)a.c	78(0.6)a	48(1.0)b	27(0.7)a.b	31(0.5)a	7(0.4)a.b	
**HPV58**	348(2.5)a	92(2.2)a.c	247(2.6)a	82(1.3)c	20(1.3)b.c	189(1.4)a	61(1.3)a	58(1.4)a	41(0.7)b	16(1.0)a.b	
**HPV59**	170(1.2)a	37(0.9)a	122(1.3)a	62(1.0)a	16(1.0)a	99(0.7)a	36(0.8)a	39(0.9)a	31(0.5)a	11(0.7)a	
**HPV66**	139(1.0)a	52(1.2)a	86(0.9)a	31(0.5)b	8(0.5)a.b	74(0.5)a	32(0.7)a	30(0.7)a	14(0.2)b	8(0.5)a.b	
**HPV68**	184(1.3)a	63(1.5)a	109(1.1)a	14(0.2)b	4(0.3)b	142(1.0)a	47(1.0)a	42(1.0)a	7(0.1)b	4(0.3)b	
**HPV82**	24(0.2)a	4(0.1)a	23(0.2)a	15(0.2)a	5(0.3)a	12(0.1)a	6(0.1)a.b	4(0.1)a.b	2(0.0)a	4(0.3)b	
**HPV6**	119(0.8)a.b	41(1.0)a.b	104(1.1)b	37(0.6)a	6(0.4)a.b	52(0.4)a	15(0.3)a	26(0.6)b	12(0.2)a	3(0.2)a	
**HPV11**	79(0.6)a	20(0.5)a	52(0.5)a	19(0.3)a	8(0.5)a	16(0.1)a	9(0.2)a	11(0.3)a	5(0.1)a	5(0.3)a	
**HPV42**	199(1.4)a	74(1.8)a	91(0.9)b	6(0.1)c	5(0.3)b.c	162(1.2)a	74(1.6)a	49(1.2)a	2(0.0)b	2(0.1)b	
**HPV43**	198(1.4)a	51(1.2)a.b	102(1.1)a.b	47(0.8)b	8(0.5)b	128(0.9)a	38(0.8)a	48(1.2)a	20(0.3)b	11(0.7)a.b	
**HPV81**	339(2.4)a	113(2.7)a	162(1.7)b	48(0.8)c	13(0.8)b.c	267(1.9)a	92(1.9)a	103(2.5)a	27(0.5)b	8(0.5)b	
**HPV83**	20(0.1)a	10(0.2)a	28(0.3)a	6(0.1)a	3(0.2)a	15(0.1)a	7(0.1)a	4(0.1)a	7(0.1)a	1(0.1)a	

Underlining indicates that the prevalence of genotypes decreased from 2018–2023

*Each subscript letter (a, b, c, d) denotes a subset of year categories whose column proportions do not differ significantly from each other at the 0.05 level

## Discussion

There were numerous studies about the prevalence of HPV infection before the COVID-19 pandemic, and they found that HPV infection and genotype distributions are regional. Furthermore, within the same region, the HPV infection rate is also affected by the sample source (clinic-base vs. population-base). In this study, we analyzed the prevalence of HPV infection and genotype distribution among two groups of women, a healthy group and an outpatient group, during the COVID-19 pandemic in Central Fujian, China. The results showed that the overall HPV infection rate was 16.1%. The infection rate in the patient group was greater than that in the healthy group (19.0% vs. 12.3%). These results are consistent with those reported in Beijing and Zhejiang [9, 10]. These findings may be related to the differences in the prevalence of HPV between the two groups: the healthy group underwent physical examination, and most of the subjects in the patient group were seeking medical intervention, such as gynecopathies. The general infection rates in this study were lower than those in previous surveys in Central Fujian (38.3%, χ2 = 2169.7, *P* < 0.0005) [11], Zhejiang (22.3%, χ2 = 600.96, *P* < 0.001) [10], and Shanghai (Putuo Hospital) (18.8%, χ2 = 76.9, *P* < 0.005) [12] but greater than that in Xi’an (13.5%, χ2 = 52.8, *P* < 0.01) [13] and Shandong (16.4%, χ2 = 1.2, *P* > 0.1) [14]. The percentage in the healthy group was greater than that in the Putian group (9.6%, χ2 = 92.52, *P* < 0.005) [15] and lower than that in the Zhejiang group (13.6%, χ2 = 15.8, *P* < 0.025) [10], Fujian group (a cross-sectional study) (16.2%, χ2 = 24.3, *P* < 0.025) [16], and Beijing group (11.9%, χ2 = 0.16, *P* > 0.1) [9]. The results for the patient group were not significantly different from those for Henan (19.7%, χ2 = 3.91, *P* > 0.05) [17] or Guangxi (18.1%, χ2 = 6.3, *P* > 0.05) [18] but were lower than those for Beijing (21.0%, χ2 = 20.4, *P* < 0.025) [19] and Zhejiang (27.3%, χ2 = 587.8, *P* < 0.001) [10]. The statistics presented support the viewpoint that the HPV infection rate varies among different regions, and this variation may be related to factors such as geographical variations; sample sources; HPV genotypes; and the local economy, living environment, and habits [19, 20]. It is noteworthy that the infection rate of HPV in Central Fujian is relatively low compared to the national average.

More than 200 HPV genotypes have been identified, with HPV16 and HPV18 thought to be responsible for approximately 70% of cervical cancer and precancerous lesions [21]. This study identified HPV52 as the most prevalent genotype, followed by HPV53, HPV58, HPV16, and HPV51, with similar rankings observed in both the healthy and patient groups. A survey conducted in Putian, Fujian, in 2022 revealed that the five most prevalent genotypes were HPV52, HPV58, HPV16, HPV18, and HPV33 [15]. These findings are significant because they demonstrate the variability in HPV genotypes even within the same province. Furthermore, compared to a previous study in Central Fujian from 2009–2015 [11], we observed a decrease in the prevalence of HPV16 during the pandemic.

This study revealed that, with the exception of six genotypes, the infection rate of most genotypes was greater in the patient group than in the healthy group. These findings underscore the importance of understanding the distribution of HPV genotypes and their associations with different patient populations. However, despite the availability of a corresponding low-risk genotype, the observation of a higher infection rate of the low-risk genotype HPV6.11 in the patient group raises important questions about the effectiveness of current vaccination strategies and the potential impact of the pandemic on vaccination rates.

The observation that the patient group had higher rates of both single and multiple HPV infections than did the healthy group is noteworthy. Furthermore, the finding that the proportions of different infection types within the overall infected population were similar between the two groups is also noteworthy. This suggests that, while the overall distribution of infection types was similar, the patient group was more susceptible to both single and multiple HPV infections. Given that multiple and persistent HPV infections are positively associated with high-grade squamous intraepithelial lesions (HSILs) [22, 23], these findings underscore the importance of closely monitoring multiple infections, particularly in the context of disease progression and potential long-term health outcomes.

The use of pairwise chi-square tests to analyze the prevalence of HPV across different age groups has provided valuable insights into the distribution of HPV infections in the study population. The identification of two peaks of HPV infection among the two age groups (≤ 24 and ≥ 55) is consistent with previous national surveys in China [24, 25]. The observed high HPV infection rates in younger individuals (≤ 24) may be attributed to factors such as frequent sexual activity and immature immune responses [26], which are known to increase susceptibility to HPV infections. Conversely, the higher incidence of HPV infections in older individuals (≥ 55) may be influenced by declining immune function and hormonal changes associated with perimenopause [27].

The relatively low participation rate of individuals aged ≤ 24 and ≥ 55 years may be indicative of cultural or behavioral factors, such as hesitancy toward gynecological examinations, especially in asymptomatic patients. This underscores the significance of overcoming health care access obstacles and advocating for routine screenings for HPV and associated gynecological conditions across all age brackets.

Moreover, the distinct distribution of HPV genotypes among various age groups, with specific genotypes exhibiting higher prevalence in younger populations, emphasizes the necessity for tailored vaccination approaches. Identifying HPV16, 51, 59, 82, 6, and 11 as more prevalent among patients aged ≤ 24 years and HPV18 and 52 as more prevalent among the healthy cohort offers crucial insights for directing vaccination initiatives. Our results suggest that the prevalence of high-risk HPV52, 53, 58, and 51, as well as low-risk HPV81, 42, and 43, was widespread across all age categories. Remarkably, the predominant low-risk variants detected in patients aged ≤ 24 years were HPV6 and HPV11, consistent with earlier research in Shandong [14]. Considering the association of HPV6 and HPV11 with the most common warts and verruca acuminate [28], prioritizing HPV vaccination with 4-valent and 9-valent vaccines for younger individuals postepidemic is crucial. Moreover, enhancing HPV surveillance and formulating vaccines tailored for elderly individuals are equally critical areas deserving attention.

The data of the subjects included in the study for each specific year were collected between 2018 and 2023. The results showed a decrease in the overall prevalence of HPV infection from 19.4 to 16.1% (χ2 = 124.5, *P* < 0.005). Specifically, the prevalence decreased from 19.0 to 23.0% (χ2 = 89.6, *P* < 0.005) in the outpatient group and from 15.7 to 12.3% (χ2 = 72.3, *P* < 0.005) in the healthy group. Furthermore, the infection rate of HPV showed a decreasing trend over time, which was particularly noticeable during the years 2022–2023. This decrease in HPV incidence can be attributed to the implementation of stringent public health policies, such as strict lockdowns, antimigration measures, and restrictions on social interactions, in response to the COVID-19 pandemic. These policies significantly altered human activities, reducing close contact between individuals and thereby lowering the risk of HPV transmission. In addition, the economic disruptions caused by the lockdowns, the reallocation of health care resources toward COVID-19 response efforts, and individuals’ heightened concerns about contracting COVID-19 may have made it more challenging for people to access medical services, including HPV screening, potentially impacting the management of HPV infection rates.

Since HPV is a primary cause of cervical cancer, a decrease in HPV incidence could have a positive impact on the prevention and control of cervical cancer in central Fujian Province and globally. First, a decrease in HPV incidence may lead to a lower incidence of cervical cancer. Second, a decrease in HPV incidence may lead to a reduced need for cervical cancer screening, thereby easing the burden on the health care system and enabling more efficient resource allocation. Third, a decrease in HPV incidence may enhance awareness and acceptance of cervical cancer vaccination, leading to a further reduction in the incidence of cervical cancer. Finally, a decrease in HPV incidence may prompt local and global health authorities to enhance monitoring, prevention, and control measures for cervical cancer, thereby offering more effective support for cervical cancer prevention and treatment.

However, there are also critical aspects that require vigilance. Analysis of the results from both groups indicated minimal changes in the infection rate and the prevalence of the most common genotypes. Moreover, the COVID-19 pandemic likely disrupted the supply chain and vaccination schedules for HPV vaccines, leading to missed vaccination opportunities for some individuals and impacting the coverage rates of vaccination programs [29, 30]. Additionally, nonpharmaceutical interventions for HPV transmission likely led to reduced access to health services, resulting in an elevated risk of undiagnosed HPV [31]. However, as the pandemic subsides and social activities gradually resume, coupled with reduced vaccination rates during this period [32], there is a potential surge in HPV infection rates [33]. Therefore, it is crucial to promptly enhance HPV screening and vaccination strategies to mitigate the potential surge in HPV dissemination.

There are several limitations to consider in this study. First, the data were collected from individuals undergoing HPV screening in hospitals, and due to the limited sample size, the results may not fully capture the epidemiological characteristics of HPV in the broader region. Future studies will aim to broaden the study population to include individuals such as low-income individuals who have not previously had access to HPV screening. Second, the HPV detection kit used in this study could only identify 22 genotypes. While it covers most types prevalent in Asian populations, there remains a risk of missing the detection of other genotypes. Finally, this was a retrospective analysis, and the lack of detailed information on the geographic, sociodemographic, and lifestyle habits of the study populations may have impacted the assessment of the effects of environmental factors on HPV infection rates. Therefore, future studies will incorporate longitudinal designs to address these limitations.

## Data Availability

No datasets were generated or analysed during the current study.

## Abbreviations

COVID-19: Coronavirus disease 2019
HPV: Human Papillomavirus
HR-HPV: High-risk human papillomavirus
LR-HPV: Low-risk human papillomavirus
HSIL: High-grade squamous intraepithelial lesions
